# HBO treatment enhances motor function and modulates pain development after sciatic nerve injury via protection the mitochondrial function

**DOI:** 10.1186/s12967-023-04414-x

**Published:** 2023-08-15

**Authors:** Yaseen Awad-Igbaria, Nadine Ferreira, Ali Keadan, Reem Sakas, Doron Edelman, Alon Shamir, Jean Francous-Soustiel, Eilam Palzur

**Affiliations:** 1 Azrieli Faculty of Medicine, Bar-Ilan University, Zefat, Israel; 2 Research Institute of Galilee Medical Center, P.O.B 21, 22100 Nahariya, Israel; 3 Psychobiology Research Laboratory, Mazor Mental Health Center, Akko, Israel; 4 UHN—Neurosurgery Spine Program, Toronto Western Hospital, University of Toronto, Toronto, ON Canada; 5 Ruth and Bruce Rappaport Faculty of Medicine, Technion—Israel Institute of Technology, Haifa, Israel; 6 Department of Neurosurgery, Galilee Medical Center, Nahariya, Israel

**Keywords:** Neuropathic pain, Neuroinflammation, Neuromodulation, Apoptosis, Mitochondrial respiration, Hyperbaric oxygen therapy (HBOT)

## Abstract

**Background:**

Peripheral nerve injury can cause neuroinflammation and neuromodulation that lead to mitochondrial dysfunction and neuronal apoptosis in the dorsal root ganglion (DRG) and spinal cord, contributing to neuropathic pain and motor dysfunction. Hyperbaric oxygen therapy (HBOT) has been suggested as a potential therapeutic tool for neuropathic pain and nerve injury. However, the specific cellular and molecular mechanism by which HBOT modulates the development of neuropathic pain and motor dysfunction through mitochondrial protection is still unclear.

**Methods:**

Mechanical and thermal allodynia and motor function were measured in rats following sciatic nerve crush (SNC). The HBO treatment (2.5 ATA) was performed 4 h after SNC and twice daily (12 h intervals) for seven consecutive days. To assess mitochondrial function in the spinal cord (L2–L6), high-resolution respirometry was measured on day 7 using the OROBOROS-O2k. In addition, RT-PCR and Immunohistochemistry were performed at the end of the experiment to assess neuroinflammation, neuromodulation, and apoptosis in the DRG (L3–L6) and spinal cord (L2–L6).

**Results:**

HBOT during the early phase of the SNC alleviates mechanical and thermal hypersensitivity and motor dysfunction. Moreover, HBOT modulates neuroinflammation, neuromodulation, mitochondrial stress, and apoptosis in the DRG and spinal cord. Thus, we found a significant reduction in the presence of macrophages/microglia and MMP-9 expression, as well as the transcription of pro-inflammatory cytokines (TNFa, IL-6, IL-1b) in the DRG and (IL6) in the spinal cord of the SNC group that was treated with HBOT compared to the untreated group. Notable, the overexpression of the TRPV1 channel, which has a high Ca^2+^ permeability, was reduced along with the apoptosis marker (cleaved-Caspase3) and mitochondrial stress marker (TSPO) in the DRG and spinal cord of the HBOT group. Additionally, HBOT prevents the reduction in mitochondrial respiration, including non-phosphorylation state, ATP-linked respiration, and maximal mitochondrial respiration in the spinal cord after SNC.

**Conclusion:**

Mitochondrial dysfunction in peripheral neuropathic pain was found to be mediated by neuroinflammation and neuromodulation. Strikingly, our findings indicate that HBOT during the critical period of the nerve injury modulates the transition from acute to chronic pain via reducing neuroinflammation and protecting mitochondrial function, consequently preventing neuronal apoptosis in the DRG and spinal cord.

**Supplementary Information:**

The online version contains supplementary material available at 10.1186/s12967-023-04414-x.

## Background

In 2016, during the Euro final in Paris, Cristiano Ronaldo suffered a devastating knee injury and pain that forced him to withdraw during the game. To speed recovery, and more importantly to relieve pain, Cristiano received intensive Hyperbaric Oxygen Therapy (HBOT), the treatment has shown a positive result in relieving pain and enhancing the recovery process [1, 2]. Novel approaches (like: HBOT) that stop the mechanisms driving the transition from acute to chronic pain can be a highly effective therapeutic tool [3]. However, the molecular and cellular mechanism by which HBOT reduces pain and improves motor function remains unclear.

Peripheral neuropathic pain (PNP) remains one of the leading causes of global disability, affecting around 10% of the population [4]. PNP is a common, complex, and distressing problem that profoundly impacts individuals and society [5]. PNP can result from nerve injury/damage, and mainly manifest as spontaneous pain, allodynia, and hyperalgesia that develop due to metabolic abnormalities, trauma, infection, and inflammation [6]. Furthermore, neuronal apoptosis of peripheral and central nociceptive pathways was found to be involved in the development of allodynia and hyperalgesia [7, 8]. Previous evidence suggests that the disruption of intracellular Ca^2+^ due to the injury is a key event in initiating the domino effect of neuronal apoptosis in the DRG neurons and spinal cord [9, 10].

The massive influx of Ca^2+^ into the injured axon contributes to neuronal apoptosis in the DRG [11]. In addition, the increased nociceptive signal from the injured nerve probably leads to the amplified synaptic release of glutamate, Substance P (SP), and calcitonin gene-related peptide (CGRP) in the spinal cord triggering disruption of intracellular Ca^2+^ in neurons, hyperexcitability, secretion of pro-inflammatory cytokines (TNFa; IL6; IL1-b), which eventually induce neuroinflammation, and neuronal apoptosis [12, 13]. The uncontrolled Ca^2+^ influx probably leads to mitochondrial membrane permeability, where calcium accumulates in the mitochondrial matrix, resulting in energy crisis (ATP depletion), cytochrome-c (Cyt-c) release, generation of free radicals and reactive oxygen species (ROS), and consequently, activation of the apoptosis pathway [14, 15]. Previous studies have shown that after sciatic nerve injury there is a significant increase in the expression of the outer mitochondrial membrane protein translocator protein (TSPO, 18kDa) in the DRG and spinal cord [16, 17]. The TSPO involves in multiple cellular functions including cell proliferation, steroidogenesis, mitochondrial permeability transition pore (mPTP) opening, mitochondrial respiration, apoptosis and inflammation [18]. However, the role of TSPO in neuropathic pain development is not fully understood. Yet, some evidence has shown that modulation of TSPO activity contributes to reducing neuroinflammation, promoting axonal regeneration, and improving mitochondrial respiration [14, 19]. Consequently, regulating the TSPO activity during the early period of neuronal injury might provide a promising tool to modulate neuropathic pain development.

Interestingly, the elevated expression of Transient Receptor Potential vanilloid 1 (TRPV1), which has a high Ca^2+^ permeability, after nerve injury in the DRG neurons and spinal cord, suggests that this channel may be involved in the Ca^2+^ influx that damages the mitochondria and initiates neuronal apoptosis [20, 21]. Unsurprisingly, modulation of TRPV1 activity in the DRG neurons, reduced the Ca^2+^ influx, prevents the mitochondrial membrane-potential damage and decreased the levels of mitochondrial ROS after nerve injury in an animal model of neuropathic pain [10]. TRPV1 is activated by a wide range of noxious stimulants including capsaicin, temperature above 43–52 °C, acidic environments (H^+^), bradykinin, and oxidative stress [10, 22–25]. In addition, TRPV1 can be activated/modulated by proinflammatory cytokines, nerve growth factors, and neurotransmitters through signal transduction pathways such as cyclic AMP-dependent protein kinase (PKA), protein kinase C (PKC), and extracellular signal-regulated protein kinase/mitogen activated protein kinase (ERK/MAPK) [12, 25, 26].

HBOT, in which patients are exposed to 100% oxygen under increased atmospheric pressure, has beneficial effects in treating pain disorders, including inflammatory pain, fibromyalgia, and migraine [27]. In addition, previous evidence has shown that HBOT exerts neuroprotective effects (anti-apoptotic) by reducing inflammation, hypoxia, edema, oxidative burden, and downregulating TRPV1 signaling [8, 28–30]. Interestingly, the anti-apoptotic mechanisms of HBOT might involve protecting mitochondrial permeability and reducing the release of Cytochrome c (Cytc), which ultimately suppress the apoptotic pathways mediated by the mitochondria [15]. However, it is still unclear whether the protective effects of HBOT on mitochondrial integrity and function, which may prevent neuronal apoptosis, are associated with the modulation of TRPV1 overactivation and overexpression following nerve injury. Accordingly, we aimed to examine whether and how HBOT protects mitochondrial function in the spinal cord following sciatic nerve injury through modulation of neuroinflammation and neuromodulation (TRPV1 pathway).

## Materials and methods

### Animals

Male Sprague–Dawley rats (250–300 g; 10 weeks age) were used in this study. All animal procedures were approved by the University Animal Care Committee and were carried out in accordance with the National Institutes of Health Guide for the Care and Use of Laboratory Animals. During the study, animals were housed in groups of 3–4 rats in a sterilized solid bottom cage with contact bedding under controlled temperature and a 12:12 h light/dark cycle. Animals were maintained on a standard pellet diet, and water was supplied ad libitum. All efforts were made to maintain animals suffering to a minimum.

### Neuropathic pain model

The sciatic nerve crush injury model (SNC) was produced using a modified technique previously described by Savastano et al. [31]. Briefly, animals were anesthetized using 3% isoflurane in 100% oxygen within an induction chamber. Once the rodents no longer responded to an interdigital pinch, they were transferred to a temperature-controlled surgery plate, received a nose cone, and were maintained on 1.5%–2.0% isoflurane for 60 min. After induction of anesthesia, a temperature of 37 °C was maintained using an isothermal pad. Next, the surgical site was shaved and disinfected, then the right paw's great trochanter (hip bone) was located and followed the femur laterally for 1 cm. Subsequently, a 1.5 cm incision in the skin on the posterior face of the thigh was made to create a gap in the thigh muscle to expose the sciatic nerve. The exposed nerve was then crushed by applying pressure with micro-dissecting tweezers twice for 15 s (at the same place) at 90° angles relative to one another to reduce the possibility of sparing. Following the crushing procedure, the muscle and the skin were sutured, and the rat was allowed to recover from anesthesia in an individual cage. After approximately sixty minutes, all the rats regained full consciousness.

### HBO treatment

HBOT was conducted based on our previous studies [28]. A cylindrical HBO chamber (Hipertech Inc, Istanbul, Turkey) was utilized for HBOT. Rats in the HBOT group were transferred to the treatment room while in their cages for thirty minutes for habituation. The chamber was flushed with 100% oxygen for 10 min before the HBOT group was placed inside the chamber. Then, the pressure was increased at a rate of 0.1 atmospheres absolute (ATA)/minute to the desired pressure (2.5 ATA) and maintained for ninety minutes, during the treatment animal status was monitored by the experimenter. The current treatment protocol and ATA-pressure were based on previous studies [30].

### Behavioral tests and pain assessment

#### Mechanical sensitivity

Mechanical sensitivity was assessed using an electronic Von Frey (eVF) device. Rats were transferred to the testing room while in their cages for one hour for habituation. Thirty minutes before the measurement began, rats were placed in the measuring chambers (20 × 20 cm Plexiglas boxes equipped with a metallic screen-mesh floor) located 20 cm above the bench for acclimatization. An Electronic von Frey device (cat. No. 38450 Ugo Basile, Varese, Italy) was used: the withdrawal threshold was evaluated by applying a force ranging from 0 to 100 g. The punctuate stimulus was delivered to the hind paw, a positive response was defined as an immediate withdrawal of hind paw in response to stimulation, and the withdrawal threshold was automatically displayed on the screen. Five measurements were performed/collected by an observer blinded to the experimental condition for each rat.

#### Thermal sensitivity

Thermal sensitivity was assessed using a computer-controlled hot plate analgesia meter (cat. No.3515-022 Ugo Basile, Varese, Italy). The animal was placed in a Plexiglas cylinder (15.5-cm diameter, 25-cm height) with a drilled cover, where the plate can be heated up to 65 °C. For the dynamic hot plate test, first, the animals were transferred to the measurement room while still in their home cage and stayed there for one hour as a habituation to the testing room. The animals were then placed on the plate for 15 min for habituation to the instrument at a temperature of 35 °C. For the dynamic hot plate test (DHP), the plate temperature increased to 45 °C with 1 °C per min speed (r^2^ = 1). An observer blinded to the experimental condition documented hind paw lickings, escape behavior (jump), and rearing for each degree interval.

#### Open field

The open field was performed during the dark phase of the cycle and conducted under red light illumination. The open field was performed by an observer blind to the experimental condition. The tests were recorded and analyzed using a computer-controlled tracking system (EthoVision XT, version 15, Noldus Information 27 Technology, Inc., Leesburg, VA). The Open Field test consists of large square, (70 cm × 70 cm × 50 cm, L × W × H). The animals were transferred to the measurement room while in cages and stayed there for one hour to habituate them to the different environments, and then, the animals were placed in the center of the maze. The session lasted 10 min, and measurements of locomotor activity and time spent in the center/peripheral of the arena were quantified. The open field was wiped with 30% alcohol solution between trials.

#### Motor outcome

Motor coordination and balance were assessed using the Rotarod (Rat Rotarod NG, Model 47,750; Ugo Basile, Varese, Italy). Animals were trained with the Rotarod one week before SNC. In training trials, rats were placed on the rod, which rotated at a constant speed of 10 rpm. The training trial continued until the rat could stay on the rod for 60 consecutive seconds without falling, turning around, or clinging to the rod. If they fell from the rod or turned around, they were correctly placed back on it, and the timer restarted. An accelerating protocol was used in test trials, where the rotation speed increased from 4 to 40 rpm for 300s. Each trial was terminated if an animal fell, clung, rotated for two complete rotations, or remained on for more than 300s. Latency to falls was automatically recorded for each trial. The average of the three tests was calculated and used for analysis. In addition, the delayed gains/motor improvement in performance was computed as the difference in performance between sessions[32]. The training and test trials were performed by an observer blind to the experimental condition.

### Histology and microscopy

#### Tissue collection

At the end of the experiment, animals were deeply anesthetized, sacrificed, and transcardially perfused with heparinized saline, 10% sucrose in buffered saline, and 4% buffered formaldehyde. The sciatic nerve, Dorsal root ganglion (L3–L6) and spinal cord (L2–L6) were excised and fixated in 5% paraformaldehyde in phosphate buffer for one hour and then embedded in paraffin. Sections of 5 μm thickness were cut from the SN, DRG and SC with a microtome, and every 5th section was collected for analysis. The sections were then mounted on Matsunami adhesive glass slide (TM-1190 TOMO, MATSUNAMI).

#### Immunohistochemistry

Immunofluorescent staining was performed as described before [22]. Briefly, slides of the dorsal root ganglion and the spinal cord were deparaffinized and subjected to heat-induced epitope retrieval using OmniPrep [pH 9.0 (10 × ), Cat. No.: ZUC067-100, ZYTOMED SYSTEMS]. According to the manufacturer's instructions, briefly, the Coplin jar containing the slides in pre-warmed solution was placed in a water bath set to 85 °C for 20 min. Followed by 3 × wash with DDW at 85 °C and rinsed with wash buffer (Zytomed systems) using a squirt bottle. Afterward, to remove background staining, we used Background Buster (#NB306-50, INNOVEX). The slides were incubated with Background Buster for 30 min at room temperature, followed by three cycles of wash buffer for two minutes each, then incubated for one hour at room temperature in a humidity chamber with one of the antibodies: anti-TRPV1 channel-488 (1:250, cat.ACC-029-AG, Alomone labs), anti-Translocator protein (1:250, AP31284PU-N, ORIGENE), anti-cleaved caspase 3 (1:500, TA336323, ORIGENE), anti-Iba1 (1:500, NB100-2833, NOVUS), anti-MMP-9 (1:500, 10,375–2-AP, Proteintech). After the first immunoreaction, the slides were washed in 1 × PBS and incubated with the second antibody for one hour at room temperature in a humidity chamber. However, When the antibodies were primary antibodies, after the first immunoreaction, the slides were washed in 1xPBS and incubated for 1 h with a secondary antibody: Donkey anti-Goat IgG-594 (1:500, #A50-201D2, Bethyl Laboratories, Inc.), Donkey anti-rabbit IgG-594 (1:500, #A120-208D2, Bethyl Laboratories, Inc.), Goat anti-rabbit IgG-488 (1:500, #A120-501F, Bethyl Laboratories, Inc.), followed by three cycles of wash buffer for two minutes each. Then, the sections were mounted with “Flouromount G with DAPI” (eBiosciences) and incubated for 30 s, then glass cover was affixed and sealed with glue.

#### Microscopy

Microscopic observation was done using the Eclipse Ci microscope (Nikon Corp., Japan). The images of 3–5 fields were captured by the Nikon DS-Ri1 camera (Nikon Corp., Japan) with the same microscope settings and exposure time. The images were analyzed using NIS Elements analysis software. The density of Microglia/Macrophages, MMP-9, Cas3, TSPO and TRPV1 was determined by the number of positively stained. The co-localization/expression of immunoreactive (IR)-TRPV1 with Caspase3 or TSPO was determined by the percentage of IR of TRPV1 with Caspase3 or TSPO. The images were analyzed by an observer blinded to the condition.

### Gene expression analysis

At the end of the experiment, animals were deeply anesthetized, sacrificed, and transcardially perfused with heparinized saline, the spinal cord (L2–L6) or the sciatic nerve and DRG (L3-L6) was isolated and kept at – 80 °C until used. The RNA was extracted from the spinal cord and the sciatic nerve-DRG samples, by combining TRI reagent (Sigma-Aldrich, St. Louis, MO, USA) and Purelink TM kit (Thermo Fisher Scientific, Waltham, MA, USA). cDNA was prepared using High-capacity cDNA Synthesis Kit (AP-4368814, Thermo Fisher Scientific, Waltham, MA, USA) and real-time PCR was performed as described before [33, 34]. Briefly, genes were assayed in triplicates for each animal using StepOnePlus real-time PCR instrument (Applied Biosystems). The relative expression of the target gene normalized to β-actin and calculated using the ΔΔCt method. The Gene primer sequences are listed in Additional file 1: Table S1.

### Mitochondrial function

#### Mitochondria isolation from spinal cord

Mitochondria were isolated from the spinal cord (L2–L6) using a differential centrifugation method [12]. Briefly, animals were deeply anesthetized, sacrificed, and the spinal cord (L2–L6) samples were harvested and washed in MiR05 (110 mM sucrose, 60 mM K^+^-lactobionate, 0.5 mM EGTA, 3 mM MgCl_2_, 20 mM taurine, 10 mM KH_2_ PO_4_, 20 mM HEPES adjusted to pH 7.1 with KOH, and 1 g/L BSA essentially fatty acid-free) for 5 min at 4 °C and then proceeded using a specially designed mitochondria isolation kit (Mito-Iso1, Sigma-Aldrich, Saint Louis, MO) according to the manufacturer instructions. Briefly, tissue samples were cut into small pieces and homogenized using a pestle and glass on ice with 10 volumes of ice-cold extraction buffer (10 m MHEPES, pH 7.5, containing 200 mM mannitol, 2 mg/mL bovine serum albumin, 70 mM sucrose, and 1 mM EGTA) and centrifuged at 600×*g* for 10 min at 4 °C. The supernatant was then collected and re-centrifuged at 11,000×*g* for 10 min at 4 °C. Pellets were resuspended with 10 volumes of a second ice-cold extraction buffer (20 mM MOPS, pH 7.5, containing 110 mM KCl and 1 mM EGTA) and processed by sequential centrifugation as above. Finally, the pellet was then resuspended in MiR05. The concentration of mitochondrial proteins was determined according to the Bradford assay using the Pierce, Coomassie Plus Protein Assay with BSA as a standard.

#### Mitochondrial respiration measurements

High-resolution respirometry was performed only on fresh tissue using the OROBOROS Oxygraph-2k (Oroboros Instruments, Innsbruck, Austria). The oxygraph was calibrated at 37 °C according to the manufacturer’s instructions with each chamber filled with 2 mL of MiR05 (110 mM sucrose, 60 mM K^+^-lactobionate, 0.5mM EGTA, 3 mM MgCl_2_, 20 mM taurine, 10 mM KH_2_ PO_4_, 20 mM HEPES adjusted to pH 7.1 with KOH, and 1 g/L BSA essentially fatty acid-free) 30 min at least before the experiment.

We used a modified protocol described by Holody et al., (2021) to evaluate mitochondrial respiration in different states [35]. The protocol evaluates mitochondrial respiration in normal state (without stimulators or inhibitors), LEAK respiration represents the non-phosphorylating state in the absence of ADP, and OXPHOS represents oxygen consumption coupled to phosphorylation of ADP to ATP in the presence of saturating ADP. The following addition steps were included: mitochondrial protein of the spinal cord (0.26–0.3 mg/mL). Leak State-non phosphorylation state through NADH-CI: pyruvate (5 mM) and malate (5 mM). OXPHOS state through ATP synthase and NADH-CI: ADP (2.5 mM), Glutamate (10 mM). OXPHOS state through ATP synthase and the combined activity of complex CI (NADH) and complex-CII (Succinate; Maximal respiration): succinate (10 mM). OXPHOS state through ATP synthase and succinate-CII**:** rotenone (0.5 µM). Leak-resting State non phosphorylation state after the activation of Succinate-CII, and inhibiting ATP synthase: oligomycin (2.5 µM). Electron transfer (ET) capacity: Uncoupler (0.5 µM). Non-mitochondrial residual oxygen consumption (ROX) after inhibition of complex III: antimycin A (2.5 µM).

Mitochondrial respiration was expressed in flux per mass (respiratory flux is expressed in pmol O_2_ per second per milligram wet tissue mass-pmol O_2_/s/mg) after correction for residual oxygen consumption (ROX), corrected automatically by DatLab^®^ software for instrumental background. The following equations were used for the calculation of the rates of oxygen consumption in a different state:

1 $${\text{Sum}}\,{\text{LEAK}}\,{\text{State}} = \left( {{\text{LEAK}}1;\,{\text{Pyruvate}}\,\& {\text{}}\,{\text{Malate}}} \right) + \left( {{\text{LEAK}}2;{\text{Oligomycin}}} \right)$$

2 $$\mathrm{Maximal\,respiration}=\left(\mathrm{ATP\,synthase};\mathrm{ADP}\right)+\left(\mathrm{OXPHOS CI};\mathrm{ Glutamate}\right)+\left(\mathrm{OXPHOS CII};\mathrm{Succinate}\right)$$

3 $$\mathrm{Sum\,OXPHOS\,State}=\left(\mathrm{ATP\,synthase};\mathrm{ADP}\right)+\left(\mathrm{OXPHOS CI};\mathrm{ Glutamate}\right)+(\mathrm{OXPHOS CII};\mathrm{Rotenone})$$

### Data analysis

Statistical analyses were performed using IBM SPSS statistics version 26 and GraphPad Prism. All data were expressed as Mean ± SEM. Differences between groups were assessed by Student *t-*test, and one-way ANOVA followed by Post-hoc Tukey’s. Changes in hypersensitivity and motor performance between tests were evaluated using mixed-model repeated-measures analysis of variance (GLM). Significant main effects and interactions were further pursued using Post-hoc by Tukey’s test and independent sample *t* test. The accepted significance value for all tests was set at *P* < 0.05.

## Results

### HBOT modulates the development of allodynia and motor dysfunction after sciatic nerve crush

**Fig. 1:**
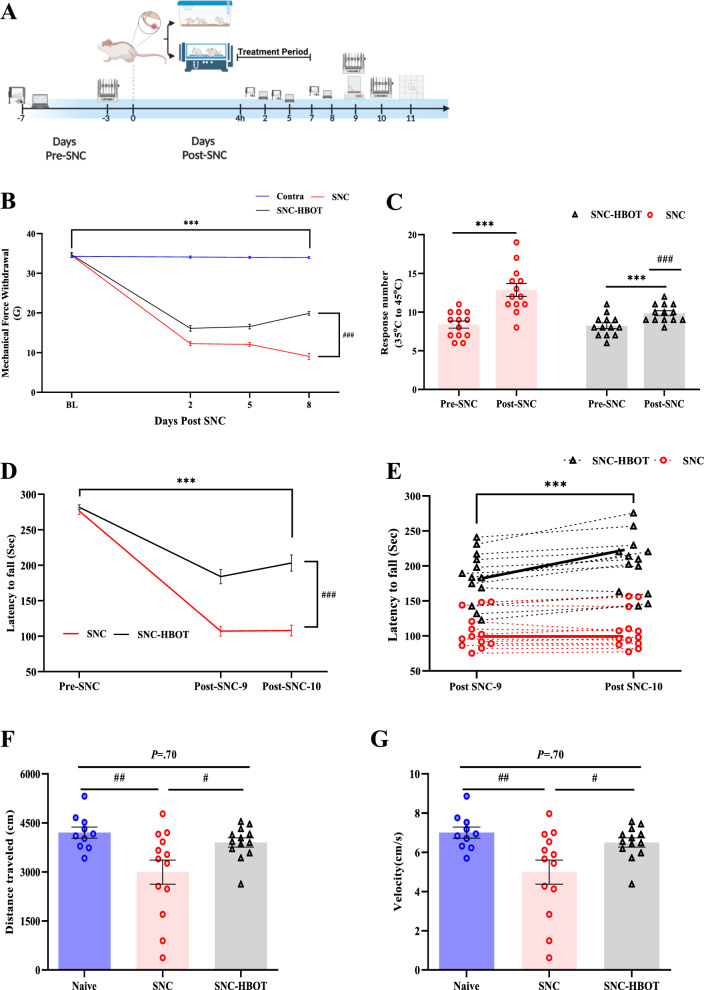
Treatment with HBO modulate the development of chronic pain after SNC. **A** The experimental timeline. Mechanical and thermal sensitivity, and motor performance were measured Pre-SNC and Post-SNC. After SNC animals were randomly assigned to one of the experiment group: SNC group, SNC-HBOT group. The HBO treatment was performed after 4h of the SNC, and twice a day to day 7 after SNC. Locomotion and motor performance tests were performed on day 9 to 11 after SNC. **B** Mechanical force withdrawal (G) of the ipsilateral and contralateral side of the SNC and the SNC^(HBOT)^ group. **C** Sum of nociceptive response in the hot plate test before and after SNC. **D** Motor performance in rotarod test expressed by latency to fall (Second). **E** Motor improvement between sessions in rotarod test expressed by latency to fall. **F** Distance moved in the OF test (cm).** G** Velocity (cm/s) in the OF test. Behavioral assessment (n = 10–13 per group). Mixed Model ANOVA-Followed with Student t-test; Parried sample t-test; Student t-test. Mean ± SEM. ^#^*P* < 0.05, ^###^*P* < 0.001 Difference between groups; ^***^P < 0.001 Compared to Baseline/Time-point comparison

Prior the SNC, there was no significant difference in mechanical and thermal sensitivity between the SNC and SNC^(HBOT)^ group [t_(24)_ = − 0.24, *P* = 0.82; t_(24)_ = 0.258, *P* = 0.79, respectively, Fig. 1B, C]. However, after the sciatic nerve crush, both groups show a significant reduction in the mechanical threshold of the injury side compared to baseline [F_(3,72)_ = 984.10, *P* < 0.001, η^2^ = 0.97, Fig. 1B], as well as a significant increase in thermal sensitivity [F_(1,24)_ = 39.92, *P* < 0.001, η^2^ = 0.62, Fig. 1C]. However, the early HBO treatment significantly modulates the mechanical and thermal hypersensitivity development (Fig. 1B, C). Thus, at each time point after the injury, the SNC^(HBOT)^ group shows higher mechanical sensitivity threshold (reduced level of allodynia, *P* < 0.05, Fig. 1B), as well as a reduced number of thermal sensitivity responses compared to the untreated group (t_(24)_ = 3.40, *P* = 0.002 Fig. 1C). The current result suggests that the early HBO treatment modulates neuropathic pain development after sciatic nerve injury.

### Neuropathic pain induces motor dysfunction

Prior the SNC, there was no significant difference in motor performance (Latency to fall) between the SNC group and SNC^(HBOT)^ group (t_(24)_ = − 0.87, *P* = 0.38, Fig. 1D). Thus, both groups show similar motor performance in the rotarod test (Fig. 1D). After the nerve crush, both groups (the SNC and the SNC^(HBOT)^) show a significant decrease in the latency to fall [F_(2,48)_ = 392.52, *P* < 0.001, η^2^ = 0.94, Fig. 1D]. However, the SNC^(HBOT)^ group shows a superior motor performance compared to the untreated group (SNC) (*P* < 0.05, Fig. 1D). In addition, after the SNC only the HBO treatment group (SNC^(HBOT)^) shows a significant improvement in performance between sessions (*P* < 0.05, Fig. 1E). Thus, approximately 90% of the rats in the SNC^(HBOT)^ group shows improvement between session, compared to approximately 50% in the SNC (Fig. 1E).

Moreover, in the open field test, we found a significant difference in the distance moved and velocity in the arena between groups [F_(2,33)_ = 5.81, *P* = 0.007; F_(2,33)_ = 5.81, *P* = 0.007, respectively, Fig. 1 F, G]. Thus, the untreated group (the SNC) traveled less distance compared to the naïve and the SNC^(HBOT)^ group (Fig. 1F). Notable no difference was found in speed and traveled distance between the naïve group and the SNC^(HBOT)^ group (*P* = 0.70, Fig. 1F, G). The current result suggests that the early HBO treatment modulates motor dysfunction after sciatic nerve injury.

### Neuroinflammation in the DRG and spinal cord after nerve injury

**Fig. 2:**
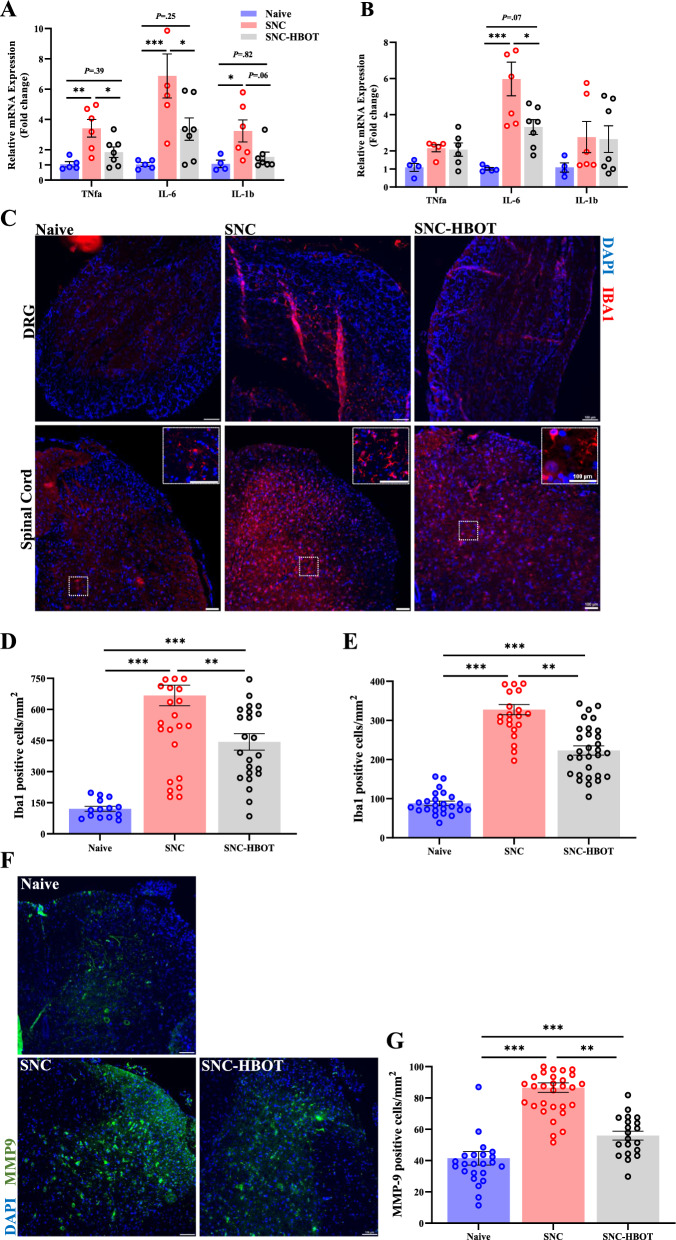
Sciatic nerve injury promotes neuroinflammation in the DRG and spinal cord. **A** Gene-expression related to inflammation in the sciatic nerve and DRG after 12 days of the sciatic nerve crush. **B** Gene-expression related to inflammation in the lumbar spine after 12 days of the sciatic nerve crush. **C** Positively stained cells with IBA1 *(Red)* and merged with dapi stain *(blue)* in the DRG and spinal cord in the naïve, SNC and SNC^(HBOT)^ group. Scale bar: 100 µm. **D** The number of positively stained cells with IBA1 in the DRG **E** The number of positively stained cells with IBA1 in the spinal cord. **F** Positively stained cells with MMP-9 *(Green)* and merged with dapi stain *(blue)* in the spinal cord in the naïve, SNC and SNC^(HBOT)^ group. Scale bar: 100 µm. **G** The number of positively stained cells with MMP-9 in the spinal cord. RT-PCR (n = 4–7), IHC (n = 4–6 animals). One-way Anova-Followed with Tukey’s test. Mean ± SEM. ^*^P < 0.05, ^**^P < 0.01, ^***^P < 0.001

Sciatic nerve injury promotes neuroinflammation in the sciatic nerve/DRG and spinal cord. Thus after 12 days of the SNC we found a significant increase in the expression of pro-inflammatory cytokines (TNF-a, IL-6, and IL-1b) in the sciatic nerve/DRG and spinal cord (Fig. 2A, B). In addition, we observed a robust increase in the presence and the activation of macrophages in the DRG, and microglia in the spinal cord after the SNC [F_(2,68)_ = 28.84, *P* < 0.001; F_(2, 75)_ = 108.87, *P* < 0.001, DRG, spinal cord, respectively, Fig. 2C–E]. Furthermore, a significant increase in the expression of Matrix metallopeptidase 9 (MMP-9) enzyme was noted in the spinal cord after the SNC [F_(2,72)_ = 84.37, *P* < 0.001, Fig. 2F and G]. However, the early HBO treatment after the nerve injury shows anti-inflammatory effects especially in the DRG. Thus, the SNC^(HBOT)^ group expressed lower level of the pro-inflammatory cytokines (TNF-a, IL-6, and IL-1b; *P* < 0.05, Fig. 2A) in the sciatic nerve/DRG, and the spinal cord (IL6; *P* < 0.05, Fig. 2B). Additionally, reduced number of macrophages/microglia was noted in the DRG and spinal cord among the SNC^(HBOT)^ group compared to the SNC group (*P* < 0.05, Fig. 2C–E). In addition, a significant reduction in the expression of MMP-9 in the spinal cord was noted in the SNC^(HBOT)^ group compared to the SNC group (*P* < 0.05, Fig. 2F, G). Together, the current result indicates that HBOT alleviates neuroinflammation induced by peripheral nerve injury.

### HBOT modulates neuromodulation-associated neuronal apoptosis

**Fig. 3:**
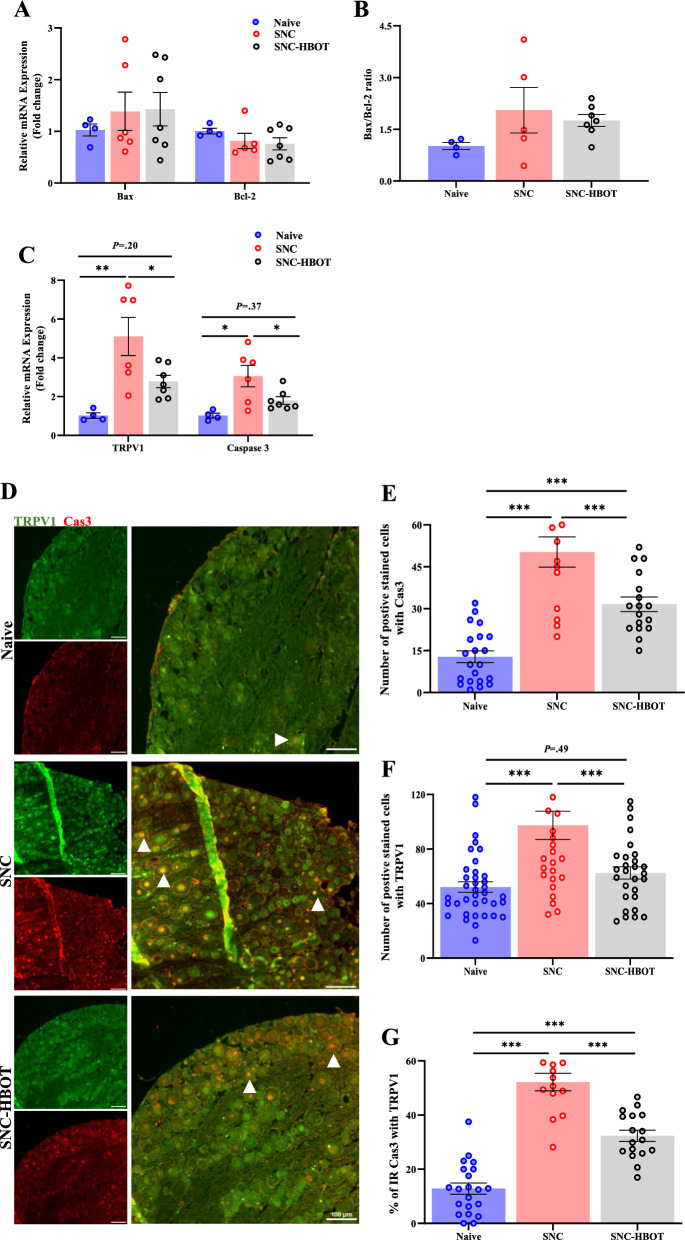
The effect of HBOT on neuronal apoptosis and neuromodulation in the DRG after nerve crush.** A** The transcription level of BAX and Bcl-2 in the sciatic nerve/DRG after 12 days of nerve crush. **B** The ratio of Bax/Bcl-2 after 12 days of nerve crush.** C** The transcription level of TRPV1 and Caspase-3 in the sciatic nerve/DRG after 12 days of nerve crush. **D** Double immunofluorescence of TRPV1 (*Green*) and cleaved Caspase-3 (*Red*), Co-localization of TRPV1 and cleaved Caspase-3 (Orange) in the DRG after 12 days of nerve crush. *scale bar: 100 µm*. **E** The number of cells that were IR-positive for cleaved Caspase-3. **F** The number of cells that were IR-positive for TRPV1. **G** The percentage of cells that were IR-positive for both TRPV1 and cleaved Caspase-3 in the DRG. RT-PCR (4–7), IHC (n = 4–6 animals). One-way Anova-Followed with Tukey’s test. Mean ± SEM. ^*^P < 0.05, ^**^P < 0.01, ^***^P < 0.001

The overexpression of TRPV1 channels might be associated with neuronal apoptosis after nerve injury [10]. Here, we found that nerve crush promotes a significant increase in the expression of TRPV1 and Caspcase-3 at the transcription level in the sciatic nerve and DRG [F_(2,14)_ = 8.78, *P* = 0.003; F_(2,14)_ = 6.79, *P* = 0.009, TRPV1, Caspcase-3, respectively, Fig. 3C]. However, the upregulated transcription level of TRPV1 and Caspcase-3 was modulated by HBOT (*P* < 0.05, Fig. 3C). Yet, no significant difference was noted in apoptosis marker BAX and Bcl2 at the transcription level in the sciatic nerve and DRG in the SNC and SNC^(HBOT)^ groups compared to the naïve group [F_(2,14)_ = 0.37, *P* = 0.69; F_(2,13)_ = 0.96, *P* = 0.40; F_(2,13)_ = 1.6, *P* = 0.23, BAX, Bcl2, BAX/Bcl2 ratio, respectively, Fig. 3A, B].

Furthermore, after the SNC we found a significant increase in the expression of TRPV1 and Caspcase-3 at the protein level in the sciatic nerve and DRG [F_(2,90)_ = 13.26, *P* < 0.001; F_(2,49)_ = 32.32, *P* < 0.001, TRPV1, Caspcase-3, respectively, Fig. 3D–F]. Additionally, we noted a significant increase in the colocalization of immunoreactivity of cleaved-Caspcase-3 with TRPV1 in the DRG among the SNC group [F_(2,49)_ = 66.46, *P* < 0.001; Fig. 3D and G]. Notable, the HBOT modulates the upregulation of Caspcase-3 activity, TRPV1 expression, and the colocalization of cleaved-Caspcase-3 with TRPV1 in the DRG compared to the untreated group (*P* < 0.05, Fig. 3D–G). However, increased level of cleaved-Caspcase-3 and the colocalization with TRPV1 was observed in the SNC^(HBOT)^ group compared to the naïve group (*P* < 0.05, Fig. 3D, E and G).

**Fig. 4:**
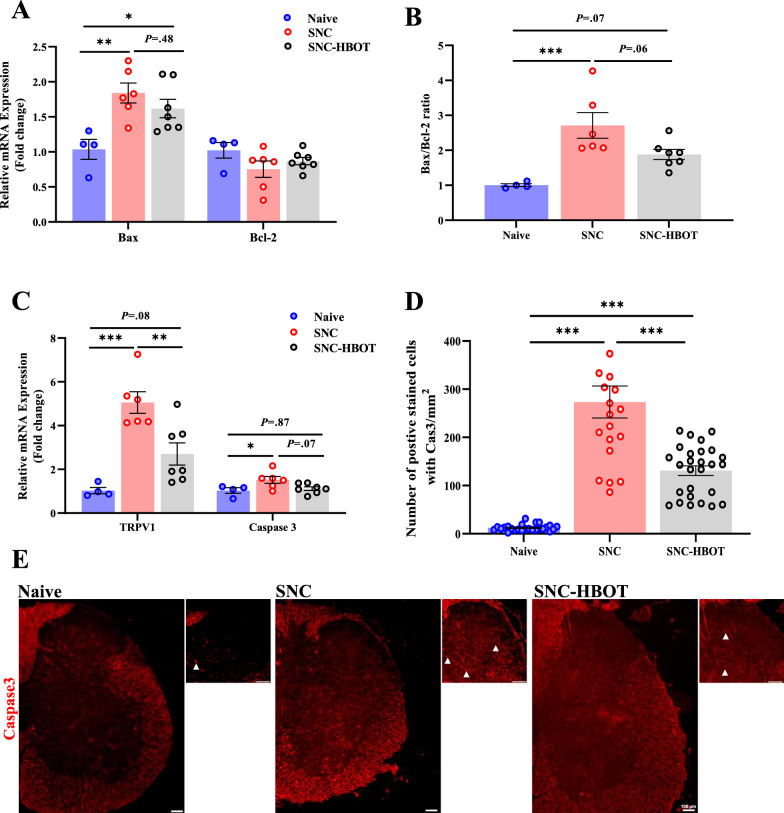
HBOT reduces neuronal apoptosis and neuromodulation in the spinal cord after nerve crush. **A** The transcription level of BAX and Bcl-2 in the spinal cord after 12 days of nerve crush. **B** The ratio of Bax/Bcl-2 in the spinal cord after nerve crush.** C** The transcription level of TRPV1 and Caspase-3 in the spinal cord after 12 days of SNC. **D** The number of cells that were IR-positive for cleaved Caspase-3 in the spinal cord. **E** Representative images of cleaved Caspase-3 in the spinal cord of the naïve, SNC and SNC^(HBOT)^ group after 12 days of nerve injury. *scale bar: 100 µm*. RT-PCR (4–7), IHC (n = 4–6 animals). One-way Anova-Followed with Tukey’s test. Mean ± SEM. ^*^P < 0.05, ^**^P < 0.01, ^***^P < 0.001

In the Spinal cord (L2–L6), we observed similar results. Thus, after 12 days of the SNC, a significant increase in the transcription of the apoptosis marker [F_(2,14)_ = 7.06, *P* = 0.008; F_(2,14)_ = 1.18, *P* = 0.19; F_(2,14)_ = 10.12, *P* = 0.002; F_(2,14)_ = 4.20, *P* = 0.03, BAX, Bcl2, BAX/Bcl2 ratio, Caspcase-3, respectively, Fig. 4A–C], and TRPV1 [F_(2,14)_ = 15.64, *P* < 0.001, Fig. 4C] in the spinal cord of the SNC group compared to the naïve group. In addition, at the protein level, we observed a robust increase in the expression of the cleaved-Caspcase-3 in the SNC group compared to the naïve group [F_(2,69)_ = 53.44, *P* < 0.001, Fig. 4D, E]. The HBOT modulates the increased transcription of TRPV1 (*P* < 0.05, Fig. 4A), but not the apoptosis marker in the spinal cord. However, the SNC^(HBOT)^ group expressed reduced level of BAX, and Caspcase-3 compared to the SNC group (Fig. 4A–C). Notable, at the protein level, decreased levels of cleaved-Caspcase-3 were found in the spinal cord of the SNC^(HBOT)^ group compared to the SNC group (*P* < 0.05, Fig. 4D, E).

### Mitochondrial distress after sciatic nerve crush

**Fig. 5:**
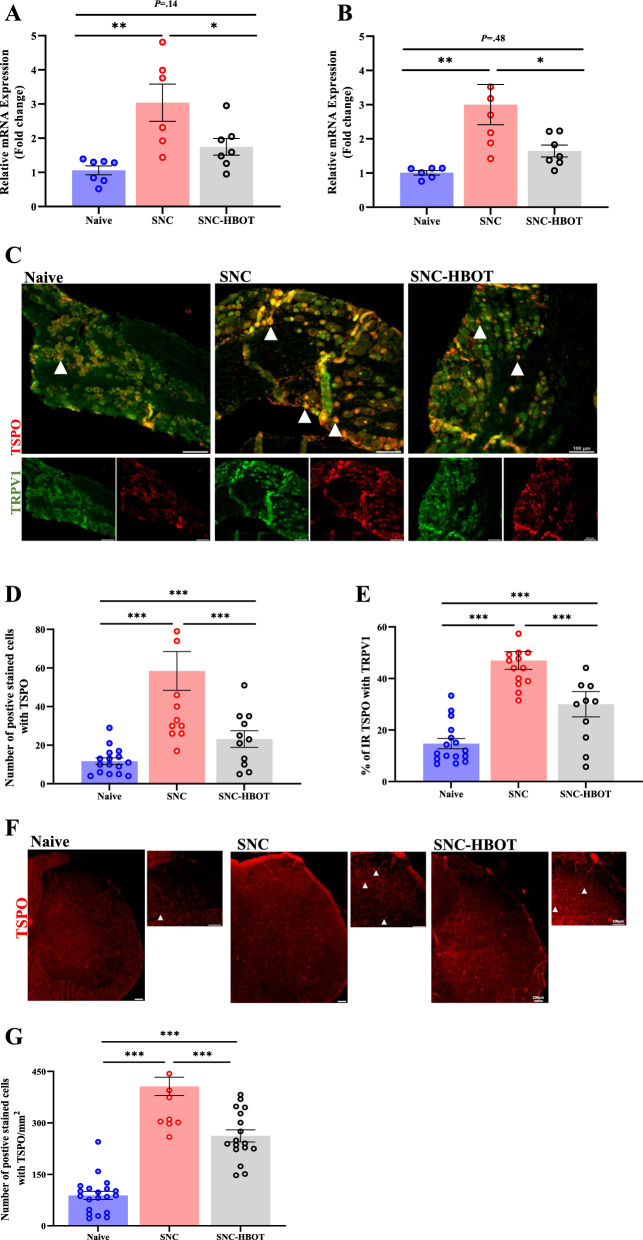
HBOT modulates mitochondrial stress after sciatic nerve injury. **A** The transcription level of mitochondria protein TSPO in the sciatic nerve-DRG after 12 days of nerve injury. **B** The transcription level of mitochondria protein TSPO in the spinal cord after 12 days of nerve injury. **C** Double immunofluorescence of TRPV1 (*Green*) and TSPO (*Red*), Co-localization of TRPV1 and TSPO (Orange) in the DRG after 12 days of nerve crush. *scale bar: 100 µm*. **D** The number of cells that were IR-positive for TSPO in the DRG. **E** The percentage of cells that were IR-positive for both TRPV1 and TSPO in the DRG.** F** Representative images of TSPO in the spinal cord of the naïve, SNC and SNC^(HBOT)^ group after 12 days of nerve injury. *scale bar: 100 µm*. The number of cells that were IR-positive for TSPO in the spinal cord. RT-PCR (4–7), IHC (n = 4–6 animals). One-way Anova-Followed with Tukey’s test. Mean ± SEM. ^*^P < 0.05, ^**^P < 0.01, ^***^P < 0.001

Here, we found that after SNC there was a significant increase in the expression of the translocator protein (TSPO) at the transcription and the protein level in the DRG [F_(2,17)_ = 9.00, *P* = 0.002; F_(2,38)_ = 15.41, *P* < 0.001, respectively, Fig. 5A, C and D]. In addition, we noted an increase in the co-expression of TSPO with TRPV1 in the DRG among the SNC group compared to the naïve group [F_(2,38)_ = 25.48, *P* < 0.001; Fig. 5C and E]. Notable, the HBO treatment downregulates the elevated expression level of the TSPO (*P* < 0.05, Fig. 5A, C and E). Thus, the SNC^(HBOT)^ group expressed lower level of TSPO at the transcription and protein level in the DRG compared to the SNC group (*P* < 0.05, Fig. 5A, C and E). Yet, at the protein level the SNC^(HBOT)^ group expressed a higher level of TSPO, and an elevated level of co-expression of TSPO with TRPV1 in the DRG compared to the naïve group.

Moreover, in the spinal cord we found that sciatic nerve injury increased the expression of TSPO at the transcription and the protein level in the SNC group compared to the naïve group [F_(2,17)_ = 7.27, *P* = 0.005; F_(2,48)_ = 75.86, *P* < 0.001, mRNA, protein, respectively, Fig. 5B, F and G]. Notable, the HBOT modulates the overexpression of the TSPO at the transcription and the protein level. Thus, reduced levels of TSPO expression were noted in the SNC^(HBOT)^ group compared to the SNC group (*P* < 0.05, Fig. 5B, F and G). However, at the protein level the SNC^(HBOT)^ group expressed higher level of TSPO in the spinal cord compared to the naïve group (*P* < 0.05, Fig. 5F and G).

### Mitochondrial respiration dysfunction in the spinal cord after sciatic nerve crush

**Fig. 6:**
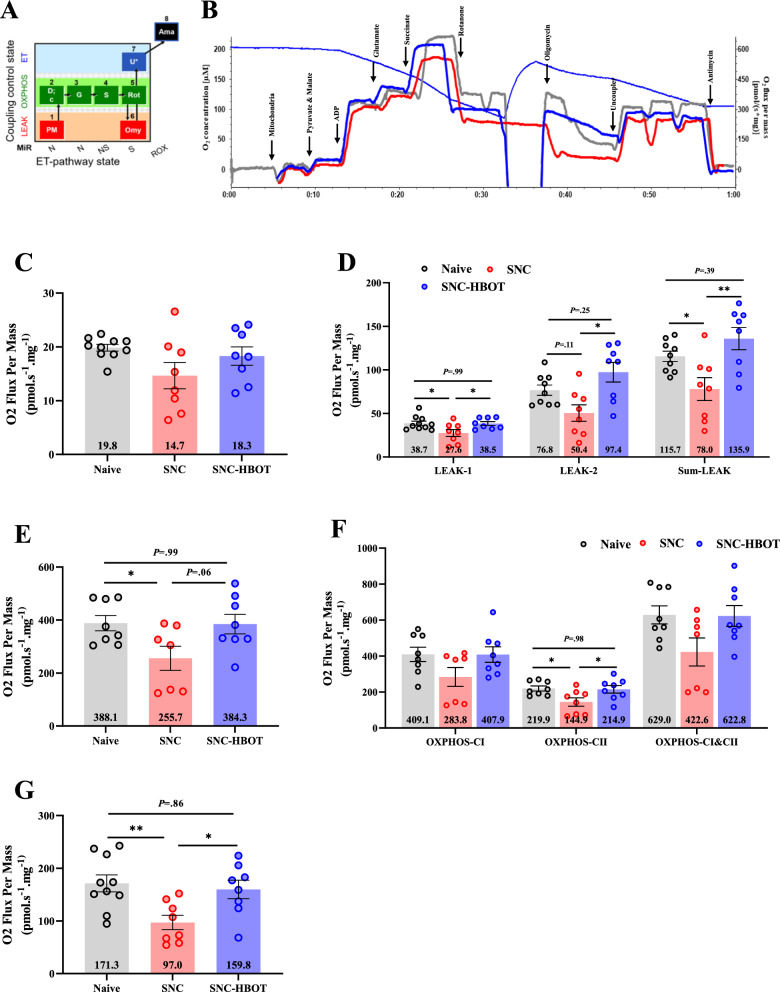
The effect of SNC on oxygen consumption in the spinal cord. **A** Protocol steps and respiratory state that were examined. The protocol examined three mitochondrial respiration states (LEAK, OXPHOS, and ET) by stimulation/ inhibition complex-I (NADH), complex-II (Succinate), and ATP synthase. Pyruvate & Malate (PM), ADP (D), Glutamate (G), Succinate (S), Rotenone (Rot), Oligomycin (Omy), Uncoupler (U), Antimycin (Ama). **B** Representative respirometric traces from spinal cord tissue using a substrate-uncoupler-inhibition protocol to measure oxygen consumption of the Naïve group (Gray line), the SNC group (Red line), the SNC^(HBOT)^ group (Blue line) after 12 days of sciatic nerve injury. Blue-Thin line represents the oxygen concentration of the chamber (left y axis) and the oxygen flux per mass (right y axis). **C** Basal mitochondrial respiration in normal state (without stimulators or inhibitors). **D** Mitochondrial respiration in the non-phosphorylation resting states-LEAK. **E** Maximal mitochondrial respiration (CI + CII). **F** Mitochondrial respiration in the OXPHOS states coupled to phosphorylation of ADP to ATP in the presence of saturating ADP (CI, CII, CI + CII). **G** Mitochondrial respiration in the Electron transfer (ET) capacity state. (n = 7–10). One-way Anova-Followed with Tukey’s test. Mean ± SEM. ^*^P < 0.05, ^**^P < 0.01, ^***^P < 0.001

We examined mitochondrial oxygen consumption in the spinal cord (L2–L6) after 7 days of SNC, in order to evaluate the mitochondrial function in different respiration states including: Leak state, OXPOHS state and Electron transfer (ET) capacity (Fig. 6A). We found that SNC did not affect the basal respiration. Thus, no differences were found between the Naïve group, SNC and SNC^(HBOT)^ group [F_(2,23)_ = 2.64, *P* = 0.09, Fig. 6B and C]. However, the damage of the nerve injury was observed in the LEAK states, thus in the LEAK state-1 and the sum LEAK states we observed a reduced level of mitochondrial oxygen consumption in the SNC group compared to the naïve group [F_(2,23)_ = 4.43, *P* = 0.02; F_(2,22)_ = 6.61, *P* = 0.006; F_(2,22)_ = 7.13, *P* = 0.004, LEAK-1, LEAK-2, Sum LEAK, respectively, Fig. 6B and D]. Strikingly, the early HBO treatment significantly enhanced the mitochondrial oxygen consumption in the LEAK states compared to the SNC group (*P* < 0.05, Fig. 6B and D). Furthermore, the activation of both CI (NADH) and CII (Succinate), represent the maximal mitochondrial respiration show a lower level of mitochondrial oxygen consumption in the SNC group compared to the naïve group [F_(2,20)_ = 3.97, *P* = 0.03, Fig. 6B and E]. Additionally, no significant difference was observed between the SNC and the SNC^(HBOT)^ group in the maximal mitochondrial respiration. However, on average, the SNC ^(HBOT)^ group showed a normal maximal respiration compared to the SNC group (Fig. 6B and E).

Stimulation of oxidative phosphorylation states (i.e., activation of ATP-synthase) via saturating of ADP and glutamate (i.e., OXPHOS state for the NADH pathway-CI), and succinate (i.e., OXPHOS state for the succinate pathway-CII) shows a non-significant reduced level of mitochondrial oxygen consumption in the SNC group compared to the naïve and SNC^(HBOT)^ group [F_(2,20)_ = 2.46, *P* = 0.11, F_(2,20)_ = 3.43, *P* = 0.052, OXPHOS-CI, OXPHOS-CI&CII, respectively, Fig. 6B and F]. However, stimulation of the succinate pathway-CII, and uncoupling the oxidative phosphorylation states through inhibiting NADH pathway-CI revealed a significant difference between the groups in mitochondrial oxygen consumption [F_(2,21)_ = 4.51, *P* = 0.02, Fig. 6B and F]. Thus, the naïve and the SNC^(HBOT)^ groups show higher mitochondrial oxygen consumption in oxidative states than in the SNC group (Fig. 6 B and F). Finally, we found that the Electron transfer (ET) capacity of the SNC group has reduced compared to the naïve group [F_(2,23)_ = 6.08, *P* = 0.008, Fig. 6B and G]. Thus, a lower mitochondrial oxygen consumption level was observed in the SNC (*P* < 0.05, Fig. 6G). Notable, the HBO treatment improved the ET capacity compared to the SNC group (*P* < 0.05, Fig. 6B and G).

## Discussion

The intricacy of the link between mitochondrial dysfunction, neuronal apoptosis, and neuroinflammation in the development of neuropathic pain conditions has been discussed before [36]. However, the current study highlights several cellular and molecular factors in the peripheral and central nervous systems involved in developing and maintaining peripheral neuropathic pain. Thus, we found that sciatic nerve crush led to motor dysfunction, reduction in exploration behavior, and a robust increase in mechanical and thermal hypersensitivity that were found to be mediated by neuroinflammation, neuromodulation, elevated level of apoptosis, and mitochondrial dysfunction in the DRG and spinal cord.

Peripheral nerve damage leads to abnormal changes in the activity and the metabolism of the damaged neurons, resulting in motor dysfunction, allodynia, and hyperalgesia at the injured site [37]. In the current study, we found that sciatic nerve crush causes a significant increase in mechanical and thermal hypersensitivity and motor dysfunction. Notable, our result indicates that the development of neuropathic pain involved neuroinflammation in the DRG and spinal cord. Hence, we found a significant increase in the presence and the activation of macrophages/microglia, and a robust increase in the expression of pro-inflammatory cytokines in the DRG and spinal cord after 12 days of the nerve injury. Current results are in line with previous findings that have shown that nerve injury leads to the recruitment of the immune system to the injury site as well as to the DRG. This process might cause neuroinflammation via over-releasing pro-inflammatory mediators (e.g., TNF-a, IL-6, IL-1b), resulting in peripheral neuronal hypersensitivity [38, 39]. Additionally, here, we observed a significant increase in the number of microglia, the positive stained cells with MMP-9 enzyme, and elevated transcription level of pro-inflammatory cytokines (IL-6) in the spinal cord after nerve injury. Unsurprisingly, this result is compatible with previous animal and human studies [40, 41].

The dramatic changes in the immune cell activity and the neuro-immune signature of gene expression in the spinal cord that characterize animals with neuropathic pain might result from the hyperactivation of the somatosensory neurons and the over-nociceptive signal that can stimulate the release of pro-inflammatory cytokines from glial cells [15, 42] eventually resulting in reduced pain threshold [43]. Therefore, it’s possible that controlling the neuroinflammation in the DRG and spinal cord during the early phase of the nerve injury might be a potential prevention strategy for modulating motor dysfunction and neuropathic pain development [15, 42]. Our results support this hypothesis. Thus, we found that HBO treatment which has been reported to exert anti-inflammatory effects via reducing hypoxia, oxidative burden, and increasing the oxygen content in circulation and tissue perfusion [30, 44, 45] strikingly reduces the number of macrophages in the DRG, microglia and MMP-9 in the spinal cord after sciatic nerve injury. Moreover, diminished transcription levels of inflammatory cytokines in the DRG and spinal cord after nerve injury were observed in the SNC^(HBOT)^ group compared to the untreated group. These results align with previous evidence that HBO treatment decreases the expression of pro-inflammatory cytokines and enzymes (e.g., TNFa, IL-6, IL-1b, MMP-9) and enhances the expression of anti-inflammatory agents, such as IL-10, in nerve injury conditions [24, 44, 46].

As expected, the beneficial effects of reducing neuroinflammation after nerve injury were remarkably observed at the behavioral level. Consequently, the HBO treatment significantly modulates the development of neuropathic pain. Thus, we observed a lower level of mechanical and thermal allodynia/hypersensitivity among the SNC^(HBOT)^ group compared to the untreated group. Notable, in the SNC^(HBOT)^ group, we noted a significant enhancement in the mechanical allodynia after 7 days of HBO treatment.

In addition, while the motor performance of both groups (SNC; SNC^(HBOT)^) was hampered after the SNC, the SNC^(HBOT)^ shows a slight reduction in motor performance and superior performance in motor coordination and locomotor activity compared to the untreated group. More critically, the advantage of the HBO treatment was more pronounced between the motor test sessions. Indeed, only the SNC^(HBOT)^ had a significant advantage of improved performance (i.e., an increase in the latency to fall) between the testing days compared to the SNC group. Taken together, the results of the current study underscore the fact that HBO treatment has beneficial effects in preventing the development of neuropathic pain and motor dysfunction through reducing neuroinflammation after peripheral nerve injury.

Previous findings suggest that neuronal apoptosis in the DRG and spinal cord might play an essential role in the development of neuropathic pain [47]. Thus, elevated neuronal apoptosis levels are frequently reported in animal models of neuropathic pain [15, 48, 49]. In line with these reports, we observed a substantial increase in apoptosis in the DRG and spinal cord after SNC in the current study. More specifically, in the DRG and spinal cord, we found a significant increase in the expression of caspase-3 at the transcription and protein level in the SNC group. Notably, in the spinal cord of the SNC group, there was a significant increase in the expression of BAX (mRNA) and the ratio of BAX/bcl2 in the SNC group. The current results suggest that sciatic nerve injury promotes apoptosis in the DRG and spinal cord, possibly contributing to pain development and maintenance.

Interestingly, our result has shown that the elevated expression of the apoptosis marker cleaved-Caspase-3 at the protein level in the DRG was strongly associated with the overexpression of the TRPV1 channel in the SNC. Overall, after SNC, we found a significant increase in the transcription of the TRPV1 channel in the DRG and spinal cord. More importantly, in the DRG, we found a substantial increase in the expression of the TRPV1 channel at the protein level in cells that initiate the apoptosis pathway through cleaving Caspase-3. Unsurprisingly, our results are compatible with previous evidence that indicates the neuroprotective effects of HBO treatment in neuropathic pain conditions [50]. Thus, we found a reduced level of apoptosis (cleaved-Caspase-3) and TRPV1 (DRG: mRNA and protein level; Spinal cord: mRNA) expression in the DRG and spinal cord of the SNC^(HBOT)^ compared to the SNC group. Moreover, we noted a significant reduction in the co-expression of TRPV1 at the protein level in cells that initiate the apoptosis pathway through cleaving Caspase-3 in the DRG.

Previous evidence suggests that the elevated expression of the TRPV1 channel, frequently reported after peripheral nerve injury [51], plays a crucial role in promoting hyperexcitability and neuronal apoptosis in the DRG [10]. The overexpression of the TRPV1 channel probably increases the Ca^2+^ influx into the cells, which causes hyperexcitability, mitochondrial stress, and ROS production [52]. These changes contribute to cellular damage accumulation, which in turn promotes apoptosis [52]. Hence it might explain the increased level of apoptosis, TRPV1 overexpression, and the co-localization of TRPV1 in apoptotic cells that were observed in the SNC group.

Current results support the notion that the overexpression of the TRPV1 channel after nerve injury is associated with an increased level of mitochondrial stress [10]. First, we found that sciatic nerve injury furthers the expression of the TSPO in the DRG and the spinal cord. The overexpression of the mitochondrial translocator protein (TSPO) is linked to several pathological conditions like neuropathic pain, cancer, and traumatic brain injury [53, 54]. Besides being a marker for neuroinflammation, some evidence suggests that overexpression of TSPO indicates abnormal mitochondrial activity [55]. Unexpectedly, in the DRG, we found a significant increase in the co-expression of the TSPO with TRPV1. Critically, however, the HBO treatment modulates the expression of TSPO. Thus, we found a lower number of positively stained cells with TSPO in the spinal cord and DRG and a reduced level of co-expression of TSPO with TRPV1 in the DRG. Taken together, the current result raises the possibility that the TRPV1 signaling pathway in cells during neuropathic pain development affects the activity of TSPO and mitochondrial activity. This might explain previous findings showing that blocking TRPV1 in the DRG improved mitochondrial membrane depolarization after sciatic nerve crush [10]. Regarding the effect of HBO treatment, since the expression and the activity of TRPV1 and TSPO are sensitive to inflammation [56, 57], the capacity of HBO treatment to modulate the expression of TSPO and TRPV1 may be attributed to the anti-inflammatory effects of this treatment.

Following current results, we speculate that the overexpression of TRPV1 and TSPO in the spinal cord after nerve injury may promote mitochondrial dysfunction. To examine this hypothesis, we assessed the mitochondrial oxygen consumption in different respiration states after 7 days of nerve injury. The current result supports this hypothesis. Despite the normal mitochondrial respiration at the basal state in the SNC, challenging the mitochondria via stimulation/inhibition complex I, complex II, and ATP-synthase revealed that sciatic nerve injury reduces mitochondrial respiration in a resting state when electron transfer occurs without phosphorylation. In addition, the proportion of oxygen consumption specific to ATP synthase activity and complex II was reduced by nerve injury. Thus, the reduction of mitochondrial respiration was observed in states that include activation of ATP-synthase and complex II. For instance, the maximal mitochondrial respiration state (CI&CII and ATP-synthase) and oxidative phosphorylation state (CII and ATP-synthase). Additionally, reduced mitochondrial respiration was noted in the electron transfer (ET) capacity state. Notable, the HBO treatment enhanced the mitochondrial respiration in each respiration state compared to the SNC group.

The reduction in the mitochondrial oxygen consumption in the different respiration states that we observed may result from a decrease in membrane potential that probably occurs under metabolically stressful conditions (e.g., hyperexcitability, neuroinflammation) that requires high ATP demand and activation of the mitochondrial corrective mechanism to reduce ROS. Previous evidence supports our hypothesis. Indeed, a reduction in membrane potential contributing to cytochrome-c release and a change in mitochondrial respiration following sciatic nerve injury were described before [15, 58, 59]. Furthermore, the beneficial effect of HBO treatment at the early stage of nerve injury on mitochondrial function may be associated with reduced oxidative stress, inflammation, and neuromodulation after HBO treatment [28]. Thus, the HBO treatment indirectly protects mitochondrial function.

Nevertheless, it’s possible that the increased metabolism level, Ca^2+^ influx, and accumulation of cellular damage in the spinal cord as a result of the neuroinflammation, hyperactivation, and remodeling of the somatosensory neurons and the over-nociceptive signal from the injured nerve leads to losing the mitochondrial transmembrane potential resulting in energy crisis (ATP depletion), ROS production and cytochrome-c (Cyt-c) release, and TSPO overexpression. However, increasing the cellular oxygen by HBO at the injury site and in the spinal cord probably improves the electron transport chain by providing free electrons available for the electron transport chain (ETC). Additionally, the abundant oxygen supply as the final electron acceptor facilitates the efficient transfer of electrons and ATP production. More critically, as was proposed before by Soustiel, et al., (2008), the protective effect of HBO might be due to the negative regulation of the proapoptotic function of the TSPO [60]. This might explain the reduced levels of TSPO expression, and apoptosis that were observed in the SNC^(HBOT)^ compared to the untreated group.

Several limitations of our study should be acknowledged. Here, we focused on the effect of the early HBO treatment on the development of neuropathic pain in a sciatic nerve crush model, thus the results obtained in the current study may not provide information to understand the effect of HBO treatment on other types of pain conditions (e.g., inflammatory pain, idiopathic pain), or the late effect of HBO treatment on mitigating neuropathic pain long after the development phase.

Despite these limitations, our findings suggest that allodynia, hyperalgesia, and motor dysfunction induced by sciatic nerve crush is mediated by neuromodulation (TRPV1 upregulation), neuroinflammation, and elevated level of apoptosis in the DRG. To our knowledge, the current result represents the first report demonstrating the association between the TRPV1 channel, apoptosis, and neuroinflammation in neuropathic pain development. Furthermore, the current result indicates that the development of peripheral neuropathic pain is accompanied by mitochondrial dysfunction, neuroinflammation, and apoptosis in the spinal cord. Crucially, HBO treatment during the critical period of the nerve injury modulates the transition from acute to chronic pain and the development of motor dysfunction by reducing neuroinflammation, neuromodulation, and apoptosis in the DRG and the spinal cord. Additionally, the HBO treatment protects mitochondrial function in the spinal cord following sciatic nerve injury, probably via modulation of neuroinflammation and neuromodulation. HBO treatment is a non-invasive therapy and easy procedure to implement. Therefore, HBOT has an advantage over surgical intervention that has lower success ratio, and medication treatment which had a significant challenging problem such as non-specific drug delivery, stability, and the ability to cross the blood–brain barrier (BBB) [61, 62]. Taken together, the current findings underscore the fact that HBO treatment has anti-apoptotic, anti-inflammatory, and neuroprotective effects that modulate the development of neuropathic pain. Thus, our results provide scientific evidence for why CR7 was right.

### Supplementary Information

**Additional file 1: Table S1.** The gene primer sequences.

## Data Availability

The datasets included in the study and the code for statistical analysis are available from the corresponding author upon request.

## Abbreviations

CR7: Cristiano Ronald-7
PNP: Peripheral neuropathic pain
TRPV1: Transient receptor potential vanilloid-1.
ROS: Reactive oxygen species
TSPO: Translocator protein
SNC: Sciatic nerve crush
DRG: Dorsal root ganglion
CI: Complex I
CII: Complex II
ET: Electron transfer
Cyt-c: Cytochrome-c
mPTP: Mitochondrial permeability transition pore
