# Supplementary material for: HBO treatment enhances motor function and modulates pain development after sciatic nerve injury via protection the mitochondrial function
Source: J Transl Med. 2023 Aug 15;21:545. doi: 10.1186/s12967-023-04414-x (PMC10428612; doi:10.1186/s12967-023-04414-x)
Supplement: Supplementary file 1 — Additional file 1: Table S1. The gene primer sequences. [file 12967_2023_4414_MOESM1_ESM.docx]

**Table S1. The Gene primer sequences**

| **Primer name** | **F/R** | **Sequence 5' - 3'** |
| --- | --- | --- |
| TRPV1 | F | AAGGATGGAACAACGGGCTAG |
|  | R | TCCTGGTAGTGAAGATGTGGG |
| TNFa | F | TCTCATTCCTGCTCGTGGCG |
|  | R | GGTGAGGAGCACGTAGTCGG |
| IL-1b | F | AATGGACAGAACATAAGCCAACA |
|  | R | CTTCTTCTTTGGGTATTGTTTGG |
| Caspase-3 | F | CTCTTCATCATTCAGGCCTGC |
|  | R | AGTAACCGGGTGCGGTAGAGTA |
| IL-6 | F | AAGAGACTTCCAGCCAGTTGCC |
|  | R | ACTGGTCTGTTGTGGGTGGTATC |
| Bax | F | GCCTTTTTGCTACAGGGTTTCAT |
|  | R | AGCAATCATCCTCTGCAGCTC |
| Bcl2 | F | TCGGTGGGGTCATGTGTG |
|  | R | AAAGGCATCCCAGCCTCC |
| TSPO | F | CGCAATGGGAGCCTACTTTGTGCG |
|  | R | GCCAGGAGGGTTTCTGCAAG |
| B-actin | F | GACGTTGACATCCGTAAAGACC |
|  | R | CTAGGAGCCAGGGCAGTAATCT |
